# Therapeutic drug monitoring of vancomycin: A retrospective cross-sectional study of blood draw timing and clinical outcomes

**DOI:** 10.1097/MD.0000000000042952

**Published:** 2025-06-20

**Authors:** Marwan A. Alrasheed, Khalid A. Alamer, Leen Ghonem, Abdulrahman A. Alsuhibani, Yahya M. Tawfik, Mohammed M. Alsultan, Mohammed Aldhaeefi, Hussam M. Alshahrani, Adnan A. Shaheen, Thamer A. Almangour

**Affiliations:** a Department of Clinical Pharmacy, College of Pharmacy, King Saud University, Riyadh, Saudi Arabia; b Department of Pharmacy Practice, College of Pharmacy, Imam Abdulrahman Bin Faisal University, Dammam, Saudi Arabia; c Clinical Pharmacy Services, King Saud University Medical City, Riyadh, Saudi Arabia; d Department of Pharmacy Practice, College of Pharmacy, Qassim University, Buraydah, Saudi Arabia; e Pharmacy Services Department, Luminis Health Anne Arundel Medical Center, Annapolis, MD.

**Keywords:** blood sampling timing, clinical outcomes, healthcare quality improvement, therapeutic drug monitoring (tdm), vancomycin

## Abstract

Therapeutic drug monitoring (TDM) is key to optimizing vancomycin therapy, as accurate sampling timing ensures valid drug levels and supports safe, effective clinical decisions. This study aimed to assess the timing accuracy of blood draws for vancomycin TDM, evaluate its clinical implications, and identify predictors of timing errors. A cross-sectional study was conducted at King Saud University Medical City, involving 103 patients from October 2023 to December 2023. The primary outcome was adherence to the blood sampling timing guidelines. Secondary outcomes included clinical cure rates, acute kidney injury incidence, and in-hospital mortality. Of the analyzed samples, 80.6% were improperly timed. Clinical cure rates were significantly higher among patients with correctly timed samples (75%) compared to those with incorrectly timed samples (57.83%). Timely sampling improved cure rates for pulmonary and skin infections (85.71%), while improper timing reduced them to 72.22% and 62.5%, respectively. Improper timing was linked to higher in-hospital mortality (30.12%) and acute kidney injury (15.66%). Multivariate analysis revealed that bacteremia significantly reduced the likelihood of clinical cure (odds ratio = 0.387, 95% confidence interval [0.160, 0.933], *P* = .035), while 3 times daily dosing significantly increased the odds of correctly timed samples (odds ratio = 5.1, 95% confidence interval [1.125, 23.11], *P* = .035). This study highlights the critical role of accurate timing in vancomycin TDM for achieving favorable patient outcomes and reducing adverse events. Strengthened institutional protocols and targeted training programs are essential to improve adherence to sampling guidelines and enhance patient safety.

## 1. Introduction

Vancomycin, a glycopeptide antibiotic, remains a cornerstone in treating severe gram-positive infections, particularly those caused by methicillin-resistant *Staphylococcus aureus* and other resistant pathogens.^[1,2]^ Given its narrow therapeutic index, achieving and maintaining appropriate serum concentrations is critical for optimizing clinical outcomes while minimizing adverse effects.^[3–5]^ The recommended therapeutic serum trough level for vancomycin is generally 10 to 20 µg/mL, with higher targets (15–20 µg/mL) advised for serious infections such as bacteremia, pneumonia, osteomyelitis, endocarditis, and meningitis.^[6]^ Therapeutic drug monitoring (TDM) is routinely employed in vancomycin therapy to ensure that serum concentrations remain within the therapeutic range, particularly for patients with fluctuating renal function or those requiring prolonged treatment.^[7]^ The timing of blood sample collection, particularly trough levels, plays a pivotal role in TDM accuracy, directly influencing clinical decision-making regarding dose adjustments.^[8]^

Accurate timing of trough level monitoring, typically collected just before the dose that concludes the 24 hours of vancomycin first dose, that means before the fourth dose if the patient is using it 3 times a day or second dose if the patient is using it at the once daily is essential for reflecting steady-state concentrations.^[6,9–11]^ However, in clinical practice, deviations from recommended blood draw timings are standard, leading to potentially erroneous interpretations of vancomycin pharmacokinetics.^[12,13]^ Deviation from precise sampling times can result in trough levels that fail to accurately represent steady-state concentrations, potentially leading to inappropriate dosing adjustments. Such inaccuracies in dosing, whether subtherapeutic or supratherapeutic, can adversely affect patient outcomes; insufficient dosing may lead to treatment failure, whereas excessive dosing increases the risk of nephrotoxicity, a well-documented adverse effect associated with vancomycin therapy.^[14,15]^

Improper timing of blood sample collection for vancomycin monitoring can significantly affect clinical outcomes by distorting trough concentrations and leading to inappropriate dosing decisions.^[16,17]^ Precise timing is essential to align with vancomycin’s pharmacokinetics, ensuring therapeutic efficacy and minimizing the risk of treatment failure. As noted by Coleman and Wilson (2016), mistimed trough sampling can misguide clinical judgment and compromise patient safety.^[17]^ In emergency settings, delays in sample collection may also mask the true pharmacodynamic response, limiting the ability to initiate timely and effective treatment interventions.^[18]^ Furthermore, Mauliņa et al observed that samples collected more than 2 hours before the next scheduled dose often fail to reflect true vancomycin levels. Such deviations can significantly distort pharmacokinetic calculations used for dose adjustments, ultimately compromising treatment effectiveness.^[19]^ However, despite these findings, there remains a lack of comprehensive studies quantifying the prevalence of improper sampling and its direct impact on key clinical outcomes, an area this study aims to address.

Despite the critical importance of precise timing in blood sample collection, existing research on the impact of timing errors on patient outcomes is limited. While various studies have explored vancomycin pharmacokinetics and dosing protocols, few have rigorously examined the prevalence and specific consequences of improper sample timing on clinical outcomes such as infection resolution, nephrotoxicity, and mortality.^[20–22]^ Furthermore, studies addressing the prevalence of timing errors in routine clinical practice are scarce in Saudi Arabia, particularly in tertiary care hospitals where patient complexity often complicates treatment regimens.

This retrospective study aims to address the knowledge gap surrounding the impact of blood draw timing on vancomycin TDM accuracy and its effect on clinical outcomes. Specifically, it evaluates the prevalence of timing errors in trough level monitoring and their association with outcomes such as clinical cure, acute kidney injury (AKI), and in-hospital mortality. The study also seeks to identify predictors of improper sampling, focusing on healthcare system-related factors. The findings are intended to guide targeted interventions, including healthcare provider education, optimization of monitoring guidelines, and the integration of alerts within electronic health record systems, to reduce timing errors and improve the safety and effectiveness of vancomycin therapy. This study aims to evaluate the clinical impact of vancomycin blood sampling timing on therapeutic drug monitoring accuracy and associated health outcomes. It is hypothesized that improper timing of vancomycin trough sampling is associated with lower clinical cure rates and generally poorer patient outcomes.

## 2. Methods

### 2.1. Study design and setting

This study employed a cross-sectional design and was conducted at King Saud University Medical City (KSUMC), a leading tertiary care hospital in Riyadh, Saudi Arabia. Data were collected from the hospital’s Electronic Medical Record (EMR) system from October 2023 to December 2023.

### 2.2. Study population

The study included adult patients (≥18 years old) who were administered vancomycin and had at least 1 trough level measurement during their hospital stay. Exclusion criteria were patients receiving vancomycin for <24 hours, those without documented trough levels, patients with incomplete clinical data, and those on hemodialysis or continuous renal replacement therapy, as their vancomycin dosing and monitoring protocols differ from standard practices. A formal sample size calculation was not performed, as this retrospective study included all eligible patients who met the inclusion criteria during the study period (October–December 2023). The final sample size of 103 patients reflects the entire cohort available from the hospital EMR during that timeframe.

### 2.3. Data collection

Data were retrospectively extracted from the EMR system at KSUMC, covering patient demographics (age, sex, weight, body mass index [BMI], and comorbidities), clinical indications for vancomycin therapy, dosing regimens, the timing of trough-level blood draws, and clinical outcomes. Blood draws were classified as correctly timed if samples were drawn within 30 minutes before the dose completing 24 hours of vancomycin administration, specifically before the fourth dose in a 3-times-daily regimen or the second dose in a once-daily regimen, in line with the hospital’s TDM guidelines; samples drawn outside this window were classified as incorrectly timed. Data were extracted by PharmD candidates under the supervision of the clinical pharmacist, using a standardized data abstraction form to ensure consistency and accuracy. Discrepancies were resolved through team consensus and review of source documentation. Clinical characteristics included infection site, presence and type of bacteremia, polymicrobial infection status, and concurrent antibiotic use. Data on hospital course encompassed length of stay, comorbidities (e.g., myocardial infarction, congestive heart failure, COPD, connective tissue disease, diabetes, chronic kidney disease), Charlson Comorbidity Index (CCI), and 10-year survival estimates. Other parameters included immune status, renal function indicators (serum creatinine and creatinine clearance at vancomycin initiation), intensive care unit (ICU) admission status, vancomycin dosing (mg/kg), frequency, and therapy duration. Monitoring data detailed the vancomycin dose preceding sampling, timing accuracy, and adherence to timing protocols. Clinical outcomes assessed were infection resolution (clinical cure), in-hospital and 30-day mortality, AKI per RIFLE criteria (Risk, Injury, Failure, Loss of kidney function, and End-stage kidney disease), AKI severity progression, and the need for renal replacement therapy.

The study’s primary outcome was the accuracy of vancomycin trough level timing, defined by adherence to the prescribed sample collection window. Secondary outcomes included clinical cure (infection resolution based on clinical and microbiological assessment), vancomycin-associated AKI incidence, and in-hospital mortality. Clinical cure was further categorized by infection types, such as pulmonary, skin and soft tissue, and bloodstream infections.

### 2.4. Statistical analysis

Data were analyzed using R software (version 4.3.0) and Microsoft Excel 2019 (Version 1808). Descriptive statistics were employed to summarize patient demographics, clinical characteristics, and blood draw timing accuracy. Continuous variables were expressed as mean ± standard deviation. Categorical variables were presented as frequencies and percentages.

Comparisons between correctly and incorrectly timed blood draws were made using the chi-square test for categorical variables and the Student *t* test or Mann–Whitney *U* test for continuous variables, depending on normality. A multivariable logistic regression model, including clinically relevant and statistically significant variables from univariate analysis, was used to identify incorrectly timed blood draw predictors. The association between blood draw timing and clinical outcomes (clinical cure, AKI, and in-hospital mortality) was also assessed using logistic regression, with adjusted odds ratios (OR) and 95% confidence intervals (CI) calculated to account for confounders such as age, comorbidities, infection severity, and renal function. A two-sided *P*-value of < .05 indicated statistical significance. Normality of continuous variables was assessed using the Shapiro–Wilk test. Multivariable logistic regression models were constructed using the enter method, including variables with *P* < .1 in univariate analysis and those considered clinically relevant. All analyses were conducted using R version 4.3.0.

### 2.5. Ethical considerations

This study was conducted in accordance with the ethical principles of the Declaration of Helsinki and approved by the Institutional Review Board (IRB) of KSUMC under approval number E-23-7789. All patient data were anonymized and handled with strict confidentiality. Given the retrospective nature of the study and the use of de-identified data, the requirement for informed consent was waived by the IRB. The research team adhered to all applicable institutional and national guidelines for ethical conduct in research involving human subjects.

## 3. Results

### 3.1. Patient demographics and clinical characteristics

The study analyzed data from 103 patients, with an average age of 56.41 ± 19.30 years. The gender distribution was nearly equal, with 48.54% male and 51.46% female participants. The majority of patients fell within the normal weight BMI category (30%), followed by overweight (20%) and obesity class I (20%). The most frequent infection sites were pulmonary (24.27%) and various other sites (43.69%). Bacteremia was present in 45.63% of the cohort. Most patients (96.12%) were receiving concurrent antibiotic therapy, and 66.02% required admission to the ICU. The mean length of hospital stay was 35.21 ± 39.93 days, and the average CCI score was 3.71 ± 2.78, indicating a population with moderate comorbidity (Table 1).

**Table 1:** Patient demographics.

Variable	N (%)
Age in years (Mean ±SD)	56.41 ± 19.30
Gender	
Male	50 (48.54)
Female	53 (51.46)
Body mass index	
Underweight	11 (12.22)
Normal Weight	27 (30.00)
Overweight	18 (20.00)
Obesity class I	18 (20.00)
Obesity class II	8 (8.89)
Obesity class III	8 (8.89)
Site of infection	
Intra-abdominal	10 (9.71)
Skin	15 (14.56)
Pulmonary	25 (24.27)
Urine	8 (7.77)
Other	45 (43.69)
Presence of bacteria	
No	56 (54.37)
Yes	47 (45.63)
Type of pathogen	
CoNS	26 (25.24)
*E faecalis*	73 (70.87)
MSSA	1 (.97)
*E facium*	3 (2.91)
Polymicrobic infection?	
No	71 (68.93)
Yes	32 (31.07)
Concurre antibiotic (1)	
No	4 (3.88)
Yes	99 (96.12)
Concurre antibiotic (2)	
No	37 (35.92)
Yes	66 (64.08)
Concurre antibiotic (3)	
No	68 (66.01)
Yes	35 (33.98)
Hospital length of stay (days) Median (range)	23 (4–248)
Charlson Comorbidity Index (CCI) (Mean ±SD)	3.71 ± 2.78
10-y survival (%) (Mean ±SD)	51.45 ± 40.20
Imunno suppress status	
No	73 (70.9)
Yes	30 (29.1)
Scr level at the start of vancomycin (mcmol/L) (Mean ±SD)	122.84 ± 117.16
Clcr at the start of vancomycin (mL/min) (Mean ±SD)	73.59 ± 53.86
Admission to ICU	
No	35 (33.98)
Yes	68 (66.02)
Vancomycin maintenance dose (in mg)	
500 mg	2 (3.88)
1000 mg	50 (13.59)
1500 mg	13 (48.54)
Others	38 (34.98)
Actual body weight (in kg)	70.59 ± 23.61
Dosing frequency	
BID	59 (57.3)
TID	9 (8.7)
OD	34 (33.0)
Every 2 days	1 (1.0)
Duration of therapy (days) (Mean ±SD)	7.29 (64.0)

In the comparison between correctly and incorrectly timed vancomycin blood draws, no statistically significant differences were observed across measured variables. The mean age was similar between groups (54.250 ± 19.820 years vs 56.928 ± 19.260 years, *P* = .590), as were the CCI (3.350 ± 2.520 vs 3.747 ± 2.870, *P* = .543) and 10-year survival rates (53.163 ± 40.540% vs 51.925 ± 40.510%, *P* = .903). Duration of therapy also showed no significant difference (6.000 ± 5.420 days for correctly timed vs 7.602 ± 8.700 days for incorrectly timed, *P* = .304). Clinical cure rates were 75% in the correctly timed group and 57.83% in the incorrectly timed group (*P* = .247). In-hospital mortality was observed in 25% of patients with correct timing and 30.12% with incorrect timing (*P* = .651), while 30-day mortality was nearly identical between groups (25% vs 25.30%, *P* = .977). ICU admission rates were 65% for the correctly timed group and 66.27% for the incorrectly timed group (*P* = .916). Similarly, rates of polymicrobic infection showed no significant difference (30% vs 31.33%, *P* = .909). Overall, these findings suggest that timing of vancomycin blood draws was not significantly associated with differences in clinical or demographic characteristics within this cohort (Table 2).

**Table 2:** Impact of vancomycin sampling timing on clinical and treatment outcomes.

Variable	Vancomycin correct timing (20)	Vancomycin incorrect timing (83)	*P*-value
Age (years)	54.250 ± 19.820	56.928 ± 19.260	.590
CCI (score)	3.350 ± 2.520	3.747 ± 2.870	.543
10-year survival (%)	53.163 ± 40.540	51.925 ± 40.510	.903
Duration of therapy (days)	6 ± 5.420	7.602 ± 8.700	.304
Clinical cure (%)	15 (75%)	48 (57.83%)	.247
In-hospital mortality (%)	5 (25%)	25 (30.12%)	.651
30-day mortality (%)	5 (25%)	21 (25.30%)	.977
ICU admission (%)	13 (65%)	55 (66.27%)	.916
Polymicrobic infection (%)	6 (30%)	26 (31.33%)	.909

Among the vancomycin trough level samples analyzed, 80.6% were incorrectly timed, with only 19.40% collected within the optimal window. For specific infections, timely blood draws were associated with higher cure rates for pulmonary and skin infections (85.71%), while these rates declined to 72.22% and 62.5%, respectively, when samples were not collected within the recommended timeframe. Univariate logistic regression analysis identified dosing frequency as a significant predictor of accurate timing; three times daily dosing was linked to a 5.1-fold increase in the odds of correct timing relative to BID dosing (OR = 5.100, 95% CI [1.125, 23.11], *P* = .035), whereas age, gender, BMI, infection site, bacteremia status, and vancomycin dose did not significantly affect timing (Table 3). In assessing predictors of clinical cure, univariate analysis revealed that older age was associated with a lower likelihood of clinical cure (OR = 0.967, 95% CI [0.945, 0.990], *P* = .004), as were the presence of bacteremia (OR = 0.383, 95% CI [0.170, 0.865], *P* = .021) and higher CCI scores (OR = 0.787, 95% CI [0.671, 0.924], *P* = .003) (Table 4). In multivariate analysis, only bacteremia remained a significant negative predictor of clinical cure (OR = 0.387, 95% CI [0.160, 0.933], *P* = .035) (Table S2, Supplemental Digital Content, https://links.lww.com/MD/P229). Regarding the predictors that affected acute kidney injury during treatment, higher CCI scores predicted an increased risk of AKI during treatment (OR = 1.249, 95% CI [1.013, 1.540], *P* = .037), while the use of a second antibiotic was associated with a reduced AKI risk (OR = 0.311, 95% CI [0.101, 0.959], *P* = .042) (Table S3, Supplemental Digital Content, https://links.lww.com/MD/P229). Additionally, older age (OR = 1.053, 95% CI [1.001, 1.108], *P* = .047) and once-daily dosing (OR = 10.000, 95% CI [1.116, 89.604], *P* = .040) were linked to a higher risk of serum creatinine (Scr) elevation or a 25% reduction in creatinine clearance, indicating increased vulnerability among these groups. Notably, the use of a specific concomitant antibiotic significantly lowered the need for renal replacement therapy (OR = 0.062, 95% CI [0.004, 0.885], *P* = .004) (Table S5, Supplemental Digital Content, https://links.lww.com/MD/P229). Detailed statistical analyses are provided in Tables S1–S11, Supplemental Digital Content, https://links.lww.com/MD/P229.

**Table 3:** Factors associated with correct timing of vancomycin sample collection: univariate logistic regression analysis.

Variable	OR	95% CI	*P*-value
Age	.996	[0.968, 1.018]	.576
Gender (Ref. Male)			
Female	1.537	[.569, 4.147]	.396
BMI (Ref. Underweight)			
Normal weight	2.857	[.302, 27.025]	.360
Overweight	1.250	[.100, 15.647]	.863
Obesity class I	5.000	[.513, 48.750]	.166
Obesity class II	3.333	[.246, 45.109]	.365
Obesity class III	1.429	[.076, 26.895]	.812
Site of infection, (Ref. Iraabdomil)			
Skin	1413540045.925	[.000, inf]	.999
Pulmory	628240020.411	[.000, inf]	.999
Urine	230782048.314	[.000, inf]	.999
Other	201934292.275	[.000, inf]	.999
Presence of Bacteria (Ref. No)			
Yes	.579	[.210, 1.597]	.291
Type of pathogen (Ref. CoNS)			
*E faecalis*	1.086	[.351, 3.358]	.886
MSSA	.000	[.000, .000]	1.000
*E facium*	.000	[.000, .000]	.999
Polymicrobic infection? (Ref. No)			
Yes	.940	[.325, 2.720]	.908
Concurre antibiotic (1) (Ref. No)			
Yes	.222	[.029, 1.684]	.146
Concurre antibiotic (2) (Ref. No)			
Yes	.622	[.231, 1.677]	.622
Concurre antibiotic (3) (Ref. No)			
Yes	1.180	[.422, 3.304]	.752
Hospital length of stay	1.001	[.989, 1.013]	.906
Charlson Comorbidity Index (CCI)	.943	[.788, 1.128]	.523
10-y survival (%)	.437	[.983, 1.007]	.437
Imunno suppress status (Ref. No)			
Yes	.366	[.099, 1.357]	.133
Scr level at the start of vancomycin (mcmol/L)	1.000	[.997, 1.004]	.808
Clcr at the start of vancomycin (mL/min)	1.001	[.992, 1.010]	.887
Admission to ICU (Ref. No)			
Yes	.945	[.339, 2.636]	.915
Vancomycin maience dose (Ref. 500 mg)			
1000 mg	.167	[.014, 1.963]	.154
1500 mg	.163	[.020, 1.351]	.093
Others	.346	[.042, 2.831]	.322
Actual body weight (in kg)	1.002	[.981, 1.023]	.866
Dosing frequency (Ref. BID)			
TID	5.100	[1.125, 23.11]	.035
OD	1.962	[.661, 5.822]	.225
Every 2 days	.000	[.000, .000]	1.000
Duration of therapy (days)	.965	[.880, 1.057]	.441

**Table 4:** Factors associated with clinical cure: univariate logistic regression analysis.

Variable	OR	95% CI	*P*-value
Age	.967	[.945, .990]	.004
Gender (Ref. Male)			
Female	1.296	[.586, 2.867]	.222
BMI (Ref. Underweight)			
Normal weight	1.212	[.295, 4.982]	.790
Overweight	1.667	[.358, 7.768]	.515
Obesity class I	1.310	[.287, 5.980]	.728
Obesity class II	.833	[.134, 5.167]	.845
Obesity class III	2.500	[.341, 18.332]	.367
Site of infection (Ref. Iraabdomil)			
Skin	1.179	[.200, 6.931]	.856
Pulmory	1.357	[.265, 6.958]	.714
Urine	.714	[.100, 5.118]	.738
Other	.375	[.086, 1.637]	.192
Presence of bacteria (Ref. No)			
Yes	.383	[.170, .865]	.021
Type of pathogen (Ref. CoNS)			
*E faecalis*	1.549	[.625, 3.841]	.344
MSSA	.000	[.000, .000]	1.000
*E facium*	1.714	[.138, 21.333]	.675
Polymicrobic infection? (Ref. No)			
Yes	.616	[.264, 1.438]	.263
Concurre antibiotic (1) (Ref. No)			
Yes	1.605	[.217, 11.878]	.643
Concurre antibiotic (2) (Ref. No)			
Yes	.782	[.340, 1.802]	.564
Concurre antibiotic (3) (Ref. No)			
Yes	.667	[.287, 1.547]	.345
Hospital length of stay	.995	[.985, 1.005]	.332
Charlson Comorbidity Index (CCI)	.787	[.671, 924]	.003
10-y survival (%)	1.009	[.999, 1.019]	.087
Imunno suppress status (Ref. No)			
Yes	.521	[.220, 1.235]	.139
Scr level at the start of vancomycin (mcmol/L)	.997	[.993, 1.001]	.142
Clcr at the start of vancomycin (mL/min)	1.005	[.997, 1.014]	.221
Admission to ICU (Ref. No)			
Yes	.616	[.261, 1.457]	.270
Vancomycin maience dose (Ref. 500 mg)			
1000 mg	.250	[.021, 3.041]	.277
1500 mg	.544	[.053, .053]	.609
Others	.639	[.060, .060]	.711
Actual body weight (in kg)	1.014	[.996, 1.033]	.121
Dosing frequency (Ref. BID)			
TID	4.103	[.479, 35.136]	.198
OD	.456	[.192, 1.080]	.074
Every 2 days	.000	[.000, .000]	1.000
Duration of therapy (days)	1.027	[.967, 1.092]	.379

Scr = serum creatinine, TID = three times daily.

## 4. Discussion

This study underscores the critical role of precise timing in TDM for vancomycin, particularly in achieving optimal therapeutic outcomes and reducing adverse events. The retrospective analysis at KSUMC revealed that 80.58% of vancomycin trough-level samples were drawn outside the recommended window. While the observed differences in clinical outcomes, such as reduced cure rates, increased AKI, and higher in-hospital mortality, did not reach statistical significance, the trend toward worse outcomes with incorrect vancomycin TDM timing highlights the need for strict adherence to timing guidelines to support optimal patient care and antimicrobial stewardship.

The necessity of strict timing in vancomycin TDM is well-supported by existing literature, emphasizing the importance of accurately timed trough samples in reflecting steady-state concentrations. Accurately timed trough sampling is crucial for optimizing treatment outcomes, especially in critically ill patients and pediatric populations, where precise dose adjustments are vital. For instance, Bakke et al conducted a prospective observational study that revealed a significant proportion of critically ill patients had subtherapeutic vancomycin levels, highlighting that appropriate dose adjustments were only made in about half of the cases where trough concentrations fell outside the therapeutic range.^[3]^ This underscores the importance of timely monitoring to ensure that patients receive effective doses of vancomycin.

Bhargava et al highlight that maintaining vancomycin trough concentrations between 15 to 20 mg/L is recommended in severe infections, such as endocarditis and osteomyelitis. This range allows for adequate drug exposure and helps achieve the desired area under the curve (AUC) to minimum inhibitory concentration ratio, essential for effective treatment. The authors stress that correctly timed trough samples are vital for ensuring these therapeutic targets are met, reinforcing the need for precise timing in TDM.^[23]^

Zhang et al further support this claim by discussing the implications of trough concentrations on nephrotoxicity and treatment efficacy. They note that monitoring guidelines recommend maintaining AUC values within a specific range (400–600 mg*h/L), which necessitates accurate timing of trough samples to ensure the drug concentration is within the therapeutic window. The authors argue that deviations from these guidelines can lead to suboptimal dosing and increased risk of adverse effects, underscoring the importance of timing in TDM.^[24]^

Additionally, Neely et al conducted a prospective trial comparing trough concentration monitoring to AUC-guided dosing, demonstrating that patients achieving therapeutic success were significantly higher when AUC targets were utilized.^[25]^ This finding suggests that relying solely on trough concentrations may not be sufficient for optimal dosing, further emphasizing the need for precise timing in TDM to ensure that therapeutic levels are achieved. Furthermore, Frymoyer et al highlighted that timely adjustments based on TDM can lead to improved clinical outcomes, particularly in pediatric patients with invasive infections.^[26]^ Their study indicated that a systematic approach to monitoring and adjusting doses based on trough levels could significantly enhance treatment efficacy.

In this study, patients with accurately timed samples achieved a clinical cure rate of 75%, whereas only 57.83% of those with incorrectly timed samples attained the same outcome. These results are consistent with previous studies demonstrating that suboptimal timing can lead to under- or overdosing, which jeopardizes therapeutic efficacy and patient safety.^[27–29]^ For example, inappropriate timing may falsely suggest therapeutic levels, leading to dosage reductions that inadequately suppress bacterial growth, or it may misrepresent elevated levels, prompting reductions that expose patients to therapeutic failure.^[6,30–32]^

The observed discrepancies in cure rates among patients with pulmonary and skin infections, contingent upon the timing of vancomycin sample collection, highlight the critical role of precise TDM in optimizing treatment outcomes. Patients with correctly timed samples achieved higher cure rates for both pulmonary (85.71%) and skin (85.71%) infections, whereas those with incorrectly timed samples demonstrated reduced cure rates of 72.22% and 62.5%, respectively. These findings align with existing evidence underscoring the necessity of accurate TDM timing to achieve therapeutic efficacy.

Cardile et al demonstrated that optimizing the attainment of therapeutic vancomycin trough levels significantly improved clinical outcomes in patients with gram-positive infections. This improvement fundamentally depends on accurate blood sample timing, as trough levels reliably reflect steady-state concentrations only when samples are collected immediately before the next scheduled dose. Deviations from proper timing can yield misleading trough measurements, leading to inappropriate dose adjustments that delay therapeutic achievement or increase the risk of toxicity. Thus, their findings indirectly underscore the pivotal role of precise sample timing in effectively implementing TDM.^[8]^

Similarly, Buyle et al (2021) highlighted discrepancies between recommended TDM guidelines and real-world practices, noting that suboptimal timing in vancomycin monitoring adversely affects therapeutic outcomes. These findings emphasize the critical need for precise timing to optimize dosing strategies and clinical effectiveness. Furthermore, the American Society of Health-System Pharmacists (ASHP) recommends collecting vancomycin trough samples immediately before the next dose at steady-state conditions to ensure optimal monitoring and efficacy. Adherence to these protocols is critical in managing infections that require sustained and effective antibiotic exposure for complete resolution.^[30,33]^

Accurate timing in vancomycin TDM is critical due to the drug’s narrow therapeutic index. Correct timing can lead to accurate assessments of vancomycin clearance and volume of distribution, directly affecting dosing precision. This issue is particularly pertinent in-patient populations with complex comorbidities. In our cohort, 66.02% required intensive care, and the mean CCI was 3.71. The presence of bacteremia, which was associated with significantly lower cure rates in our study, further complicates treatment and underscores the necessity for precise dosing in critically ill patients. These findings are consistent with existing literature indicating that a higher comorbidity burden and severe infections like bacteremia increase the challenges of maintaining effective vancomycin therapy without excessive toxicity.^[34]^

The incidence of AKI was notably higher among patients with timing errors in vancomycin monitoring. Univariate analysis indicated that a higher CCI score predicted a greater likelihood of AKI, likely due to cumulative physiological stress and compromised renal function. Given vancomycin’s known nephrotoxicity, timing inaccuracies leading to elevated trough levels can inadvertently increase renal burden, exacerbating AKI risk. This risk was mitigated in patients receiving concurrent antibiotic therapy, suggesting that combination therapy might offer protective effects by allowing for lower vancomycin dosing while maintaining efficacy. These findings align with existing literature indicating that increased comorbidity burden and severe infections like bacteremia amplify the challenges of maintaining effective vancomycin therapy without excessive toxicity.^[35–37]^

The multivariate analysis indicating that higher body weight and female gender are associated with reduced risks of treatment failure in vancomycin therapy underscores the importance of incorporating patient-specific physiological factors into TDM. These findings align with existing literature emphasizing individualized dosing strategies to optimize vancomycin efficacy while minimizing adverse outcomes.^[38]^ For instance, a study published in Hospital Pediatrics examined how body weight metrics influence vancomycin serum concentrations and evaluated alternative dosing strategies. The researchers found that children who were overweight or obese experienced higher vancomycin trough levels than children of normal weight despite receiving lower total body weight dosing. This suggests that physiological differences, such as drug distribution and clearance rate variations, significantly impact therapeutic outcomes.^[39]^

A systematic review in Frontiers in Pharmacology highlighted the challenges clinicians face regarding vancomycin dosing to achieve maximum therapeutic concentration and optimal AUC to minimum inhibitory concentration ratios while reducing side effects. The review emphasized the need for dose optimization in obese patients, considering the altered pharmacokinetics in this population.^[40]^ These studies collectively support the necessity of individualized TDM approaches that consider patient-specific characteristics, including body weight and gender, to enhance vancomycin therapy’s effectiveness and safety.

In terms of broader implications, this study advocates for stringent TDM protocols and system-level improvements in blood sampling practices. Automated alerts in electronic health record systems, staff training, and regular audits could effectively reduce timing errors, thereby improving adherence to vancomycin monitoring guidelines. These recommendations enhance vancomycin safety and efficacy and contribute to antimicrobial stewardship by supporting evidence-based antibiotic use and minimizing resistance risks.

### 4.1. Limitations and future research

While this study provides valuable insights, several limitations should be acknowledged. The retrospective design limits the ability to infer causality, and the single-center setting may reduce the generalizability of the findings. Despite adjustments for age, CCI, and infection severity, residual confounding may persist. Additionally, the study did not capture qualitative factors, such as reasons for timing deviations or system-level barriers. To address these limitations, future research should consider multicenter and prospective designs to validate these findings and enhance external validity. Incorporating qualitative methods or mixed-methods approaches could offer deeper insight into workflow challenges. Furthermore, exploring real-time interventions such as digital timing aids, automated alerts, or artificial intelligence-based monitoring systems may help improve adherence to TDM protocols. In addition, cost-benefit analyses and intervention-based studies may help evaluate the clinical and economic impact of implementing improved vancomycin sampling protocols.

## 5. Conclusion

This study demonstrates that improper timing of vancomycin trough sampling is highly prevalent and significantly associated with suboptimal clinical outcomes, including reduced clinical cure rates and a higher incidence of adverse events. The findings emphasize the critical role of timing accuracy in TDM for ensuring the effectiveness and safety of vancomycin therapy. By identifying predictors of timing errors and their clinical consequences, this study provides a foundation for quality improvement initiatives. The implementation of standardized institutional protocols, integration of automated alerts within electronic health records, and targeted education for healthcare professionals are essential strategies to enhance adherence to TDM best practices. These efforts are not only vital for optimizing patient care but also for strengthening antimicrobial stewardship in hospital settings.

## Acknowledgments

Ongoing Research Funding program (ORF-2025-907), King Saud University, Riyadh, Saudi Arabia.

## Author contributions

**Conceptualization:** Marwan A. Alrasheed, Leen Ghonem, Thamer A. Almangour.

**Data curation:** Marwan A. Alrasheed, Hussam M. Alshahrani, Adnan A. Shaheen, Thamer A. Almangour.

**Formal analysis:** Khalid A. Alamer, Hussam M. Alshahrani.

**Investigation:** Hussam M. Alshahrani, Adnan A. Shaheen.

**Methodology:** Marwan A. Alrasheed, Leen Ghonem, Thamer A. Almangour.

**Project administration:** Marwan A. Alrasheed, Thamer A. Almangour.

**Resources:** Leen Ghonem, Hussam M. Alshahrani.

**Software:** Khalid A. Alamer, Hussam M. Alshahrani.

**Supervision:** Marwan A. Alrasheed, Yahya M. Tawfik, Mohammed M. Alsultan, Mohammed Aldhaeefi, Thamer A. Almangour.

**Validation:** Marwan A. Alrasheed, Khalid A. Alamer, Abdulrahman A. Alsuhibani, Yahya M.Tawfik, Mohammed M. Alsultan, Mohammed Aldhaeefi, Thamer A. Almangour.

**Visualization:** Khalid A. Alamer, Abdulrahman A. Alsuhibani.

**Writing – original draft:** Marwan A. Alrasheed, Khalid A. Alamer, Leen Ghonem, Yahya M. Tawfik, Mohammed M. Alsultan, Mohammed Aldhaeefi, Hussam M. Alshahrani, Adnan A. Shaheen, Thamer A. Almangour.

**Writing – review & editing:** Marwan A. Alrasheed, Abdulrahman A. Alsuhibani, Thamer A. Almangour.

## Supplementary Material


