# Supplementary material for: Therapeutic drug monitoring of vancomycin: A retrospective cross-sectional study of blood draw timing and clinical outcomes
Source: Medicine (Baltimore). 2025 Jun 20;104(25):e42952. doi: 10.1097/MD.0000000000042952 (PMC12187348; doi:10.1097/MD.0000000000042952)
Supplement: Supplementary file 1 [file medi-104-e42952-s001.docx]

**Table S1: Multivariate Logistic Regression (Out: Was the timing correct?)**

| **Variable** | **ODDS Ratio** | **95% CI** | **p** |
| --- | --- | --- | --- |
| **Dosing frequency (Ref. BID)** |  |  |  |
| TID | 5.100 | [1.125,23.11] | **.035** |
| OD | 1.962 | [.661,5.822] | .225 |
| Every 2 Days | .000 | [.000,.000] | 1.000 |

**Table S2: Multivariate Logistic Regression (Out: Clinical cure)**

| **Variable** | **ODDS Ratio** | **95% CI** | **P** |
| --- | --- | --- | --- |
| **Age** | .992 | [.959,1.026] | **.648** |
| **Presence of Bacteria (Ref. No)** |  |  |  |
| Yes | .387 | [.160,.933] | **.035** |
| **Charlson Comorbidity Index (CCI)** | .810 | [.638,1.029] | **.084** |

**Secondary Univariate Logistic Regression**

**Table S3: Univariate Logistic Regression (Out: Overall Acute Kidney Injury during treatment per RIFLE)**

| **Variable** | **ODDS Ratio** | **95% CI** | **p** |
| --- | --- | --- | --- |
| **Age** | 1.029 | [.997,1.061] | .074 |
| **Gender (Ref. Male)** |  |  |  |
| Female | .582 | [.191,1.773] | .314 |
| **BMI, (Ref. Underweight)** |  |  |  |
| Normal Weight | 1.000 | [.000, inf] | 1.000 |
| Overweight | 461564264.334 | [.000, inf] | .999 |
| Obesity Class I | 323094985.034 | [.000, inf] | .999 |
| Obesity Class II | 230782132.167 | [.000, inf] | .999 |
| Obesity Class III | 969284955.102 | [.000, inf] | .999 |
| **Site of infection, (Ref. Iraabdomil)** |  |  |  |
| Skin | 248534123.140 | [.000, inf] | .999 |
| Pulmory | 140475808.731 | [.000, inf] | .999 |
| Urine | 230781685.772 | [.000, inf] | .999 |
| Other | 461563371.545 | [.000, inf] | .999 |
| **Presence of Bacteria (Ref. No)** |  |  |  |
| Yes | 1.436 | [.479,4.306] | .518 |
| **Type of pathogen (Ref. CoNS)** |  |  |  |
| E. faecalis | .354 | [.107,1.173] | .089 |
| MSSA | 5384916142.837 | [.000, inf] | 1.000 |
| E. facium | 1.667 | [.128,21.732] | .697 |
| **Polymicrobic infection? (Ref. No)** |  |  |  |
| Yes | 1.130 | [.352,3.621] | .838 |
| **Concurre antibiotic (1) (Ref. No)** |  |  |  |
| Yes | 288477759.65 | [.000, inf] | .999 |
| **Concurre antibiotic (2) (Ref. No)** |  |  |  |
| Yes | .311 | [.101,.959] | **.042** |
| **Concurre antibiotic (3) (Ref. No)** |  |  |  |
| Yes | .483 | [.127,1.845] | .287 |
| **Hospital length of stay** | 1.004 | [.992,1.016] | .484 |
| **Charlson Comorbidity Index (CCI)** | 1.249 | [1.013,1.540] | **.037** |
| **10-y survival (%)** | 1.001 | [.987,1.014] | .943 |
| **Imunno suppress status (Ref. No)** |  |  |  |
| Yes | 1.260 | [.391,4.057] | .698 |
| **Scr level at the start of vancomycin (mcmol/L) ‎** | 1.000 | [.996,1.005] | .931 |
| **Clcr at the start of vancomycin (ml/min)** | 1.004 | [.994,1.014] | .417 |
| **Admission to ICU (Ref. No)** |  |  |  |
| Yes | 1.496 | [.439,5.092] | .520 |
| **Vancomycin maience dose (Ref. 500 mg)** |  |  |  |
| 1000 mg | 179497354.594 | [.000, inf] | .999 |
| 1500 mg | 1009672619.589 | [.000, inf] | .999 |
| Others | 244769119.900 | [.000, inf] | .999 |
| **Actual body weight (in kg)** | 1.025 | [1.000,1.051] | **.049** |
| **Dosing frequency (Ref. BID)** |  |  |  |
| TID | 269245770.726 | [.000, inf] | .999 |
| OD | 179497180.484 | [.000, inf] | .999 |
| Every 2 Days | 478659147.957 | [.000, inf] | .999 |
| **Duration of therapy (days)** | .976 | [.890,1.070] | .603 |

**Table S4: Multivariate Logistic Regression (Out: Overall Acute Kidney Injury during treatment per RIFLE)**

| **Variable** | **ODDS Ratio** | **95% CI** | **p** |
| --- | --- | --- | --- |
| **Concurre antibiotic (2) (Ref. No)** |  |  |  |
| Yes | .290 | [.082,1.031] | **.056** |
| **Charlson Comorbidity Index (CCI)** | 1.276 | [1.007,1.617] | **.044** |
| **Actual body weight (in kg)** | 1.025 | [.997,1.055] | **.086** |

**Secondary Univariate Logistic Regression**

**Table S5: Univariate Logistic Regression (Out: Risk (Scr increase of 1.5 times or 25% reduction in CrCL))**

| **Variable** | **ODDS Ratio** | **95% CI** | **p** |
| --- | --- | --- | --- |
| **Age** | 1.053 | [1.001,1.108] | **.047** |
| **Gender (Ref. Male)** |  |  |  |
| Female | 1.279 | [.272,6.023] | .756 |
| **BMI, (Ref. Underweight)** |  |  |  |
| Normal Weight | 1.000 | [.000, inf] | 1.000 |
| Overweight | 201934386.748 | [.000, inf] | .999 |
| Obesity Class I | 1.000 | [.000, inf] | 1.000 |
| Obesity Class II | 230782156.283 | [.000, inf] | .999 |
| Obesity Class III | 538491697.994 | [.000, inf] | .999 |
| **Site of infection, (Ref. Iraabdomil)** |  |  |  |
| Skin | 1.000 | [.000, inf] | 1.000 |
| Pulmory | 67311415.738 | [.000, inf] | .999 |
| Urine | 230781996.815 | [.000, inf] | .999 |
| Other | 201934247.213 | [.000, inf] | .999 |
| **Presence of Bacteria (Ref. No)** |  |  |  |
| Yes | .886 | [.188,4.175] | .897 |
| **Type of pathogen (Ref. CoNS)** |  |  |  |
| E. faecalis | .696 | [.120,4.043] | .686 |
| MSSA | .000 | [.000, inf] | 1.000 |
| E. facium | 6.000 | [.365, 98.720] | .210 |
| **Polymicrobic infection? (Ref. No)** |  |  |  |
| Yes | 1.733 | [.364,8.238] | .490 |
| **Concurre antibiotic (1) (Ref. No)** |  |  |  |
| Yes | 122916537.89 | [.000, inf]] | .999 |
| **Concurre antibiotic (2) (Ref. No)** |  |  |  |
| Yes | 1.434 | [.264,7.787] | .676 |
| **Concurre antibiotic (3) (Ref. No)** |  |  |  |
| Yes | .333 | [.038,2.888] | .319 |
| **Hospital length of stay** | .991 | [.962,1.021] | .555 |
| **Charlson Comorbidity Index (CCI)** | 1.246 | [.939,1.655] | .128 |
| **10-y survival (%)** | 1.007 | [.987,1.028] | .464 |
| **Imunno suppress status (Ref. No)** |  |  |  |
| Yes | .385 | [.044,3.343] | .387 |
| **Scr level at the start of vancomycin (mcmol/L) ‎** | .880 | [.992,1.007] | .880 |
| **Clcr at the start of vancomycin (ml/min)** | .987 | [.965,1.001] | .286 |
| **Admission to ICU (Ref. No)** |  |  |  |
| Yes | 3.290 | [.380,28.472] | .279 |
| **Vancomycin maience dose (Ref. 500 mg)** |  |  |  |
| 1000 mg | 1.000 | [.000, .000] | 1.000 |
| 1500 mg | 484644406.264 | [.000, .000] | .999 |
| Others | 190056629.907 | [.000, .000] | .999 |
| **Actual body weight (in kg)** | 1.027 | [.993,1.062] | .127 |
| **Dosing frequency (Ref. BID)** |  |  |  |
| TID | 7.250 | [.412,127.700] | .176 |
| OD | 10.000 | [1.116,89.604] | **.040** |
| Every 2 Days | .000 | [.000, inf] | 1.000 |
| **Duration of therapy (days)** | 1.028 | [.963,1.097] | .409 |

**Table S6: Multivariate Logistic Regression (Out: Risk (Scr increase of 1.5 times or 25% reduction in CrCL)**

| **Variable** | **ODDS Ratio** | **95% CI** | **p** |
| --- | --- | --- | --- |
| **Age** | 1.041 | [.990,1.095] | .115 |
| **Dosing frequency (Ref. BID)** |  |  |  |
| TID | 8.636 | [.460,162.241] | .150 |
| OD | 6.584 | [.669,62.909] | .102 |
| Every 2 Days | .000 | [.000,.000] | 1.000 |

**Secondary Univariate Logistic Regression**

**Table S7: Univariate Logistic Regression (Out: Injury (Scr increase of 2 times or 50% reduction in CrCL)**

| **Variable** | **ODDS Ratio** | **95% CI** | **P** |
| --- | --- | --- | --- |
| **Age** | 1.036 | [.953,1.126] | .405 |
| **Gender (Ref. Male)** |  |  |  |
| Female | .000 | [.000,.000] | .997 |
| **BMI, (Ref. Underweight)** |  |  |  |
| Normal Weight | 1.000 | [.000, inf] | 1.000 |
| Overweight | 1.000 | [.000, inf] | 1.000 |
| Obesity Class I | 1.000 | [.000, inf] | 1.000 |
| Obesity Class II | 1.000 | [.000, inf] | 1.000 |
| Obesity Class III | 230782110.781 | [.000, inf] | .999 |
| **Site of infection, (Ref. Iraabdomil)** |  |  |  |
| Skin | 1.000 | [.000, inf] | 1.000 |
| Pulmory | 1.000 | [.000, inf] | 1.000 |
| Urine | 1.000 | [.000, inf] | 1.000 |
| Other | 75138373.207 | [.000, inf] | .999 |
| **Presence of Bacteria (Ref. No)** |  |  |  |
| Yes | 1.196 | [.073,19.649] | .900 |
| **Type of pathogen (Ref. CoNS)** |  |  |  |
| E. faecalis | .347 | [.021,5.761] | .460 |
| MSSA | .000 | [.000, inf] | 1.000 |
| E. facium | .000 | [.000, inf] | .999 |
| **Polymicrobic infection? (Ref. No)** |  |  |  |
| Yes | .000 | [.000, inf] | .998 |
| **Concurre antibiotic (1) (Ref. No)** |  |  |  |
| Yes | 33308759.465 | [.000, inf] | .999 |
| **Concurre antibiotic (2) (Ref. No)** |  |  |  |
| Yes | .554 | [.034,9.112] | .679 |
| **Concurre antibiotic (3) (Ref. No)** |  |  |  |
| Yes | 2.156 | [.131,35.577] | .591 |
| **Hospital length of stay** | .976 | [.896,1.062] | .567 |
| **Charlson Comorbidity Index (CCI)** | 1.109 | [.674,1.824] | .684 |
| **10-y survival (%)** | .998 | [.964,1.034] | .930 |
| **Imunno suppress status (Ref. No)** |  |  |  |
| Yes | .000 | [.000, inf] | .998 |
| **Scr level at the start of vancomycin (mcmol/L) ‎** | 1.002 | [.994,1.010] | .615 |
| **Clcr at the start of vancomycin (ml/min)** | .957 | [.983,1.027] | .224 |
| **Admission to ICU (Ref. No)** |  |  |  |
| Yes | 489537787.291 | [.000, inf] | .998 |
| **Vancomycin maience dose (Ref. 500 mg)** |  |  |  |
| 1000 mg | 67311488.574 | [.000, inf] | .999 |
| 1500 mg | 1.000 | [.000, inf] | 1.000 |
| Others | 1.000 | [.000, inf] | 1.000 |
| **Actual body weight (in kg)** | 1.028 | [.973,1.087] | .324 |
| **Dosing frequency (Ref. BID)** |  |  |  |
| TID | 1.000 | [.000, inf] | 1.000 |
| OD | 100967174.019 | [.000, inf] | .997 |
| Every 2 Days | 1.000 | [.000, inf] | 1.000 |
| **Duration of therapy (days)** | .781 | [.404,1.509] | .462 |

**Table S8: Univariate Logistic Regression (Out: Failure (Scr increase of 3 times or 75% reduction in CrCL)**

| **Variable** | **ODDS Ratio** | **95% CI** | **P** |
| --- | --- | --- | --- |
| **Age** | 1.022 | [.982,1.063] | .292 |
| **Gender (Ref. Male)** |  |  |  |
| Female | .118 | [.014,.998] | .**050** |
| **BMI, (Ref. Underweight)** |  |  |  |
| Normal Weight | 1.000 | [.000, inf] | 1.000 |
| Overweight | 201934386.748 | [.000, inf] | .999 |
| Obesity Class I | 1.000 | [.000, inf] | 1.000 |
| Obesity Class II | 230782156.283 | [.000, inf] | .999 |
| Obesity Class III | 538491697.994 | [.000, inf] | .999 |
| **Site of infection, (Ref. Iraabdomil)** |  |  |  |
| Skin | 2483534365.797 | [.000, inf] | 1.000 |
| Pulmory | 67311390.737 | [.000, inf] | .999 |
| Urine | 1.000 | [.000, inf] | 1.000 |
| Other | 230782156.283 | [.000, inf] | .999 |
| **Presence of Bacteria (Ref. No)** |  |  |  |
| Yes | 3.951 | [.758,20.595] | .103 |
| **Type of pathogen (Ref. CoNS)** |  |  |  |
| E. faecalis | .444 | [.093,2.135] | .311 |
| MSSA | 12385307128.525 | [.000, inf] | 1.000 |
| E. facium | .000 | [.000, inf] | .999 |
| **Polymicrobic infection? (Ref. No)** |  |  |  |
| Yes | 1.366 | [.306,6.098] | .683 |
| **Concurre antibiotic (1) (Ref. No)** |  |  |  |
| Yes | 142019755.669 | [.000, inf] | .999 |
| **Concurre antibiotic (2) (Ref. No)** |  |  |  |
| Yes | .161 | [.031,.864] | **.031** |
| **Concurre antibiotic (3) (Ref. No)** |  |  |  |
| Yes | .688 | [.131,3.608] | .658 |
| **Hospital length of stay** | 1.001 | [.999,1.023] | .082 |
| **Charlson Comorbidity Index (CCI)** | 1.117 | [.862,1.447] | .402 |
| **10-y survival (%)** | .997 | [.979,1.015] | .709 |
| **Imunno suppress status (Ref. No)** |  |  |  |
| Yes | 2.654 | [.618,11.398] | .189 |
| **Scr level at the start of vancomycin (mcmol/L) ‎** | .998 | [.989,1.007] | .596 |
| **Clcr at the start of vancomycin (ml/min)** | 1.013 | [1.001,1.025] | **.030** |
| **Admission to ICU (Ref. No)** |  |  |  |
| Yes | .847 | [.190,3.769] | .827 |
| **Vancomycin maience dose (Ref. 500 mg)** |  |  |  |
| 1000 mg | 103115708.240 | [.000, inf] | .999 |
| 1500 mg | 484643828.729 | [.000, inf] | .999 |
| 1500 mg | 89748857.172 | [.000, inf] | .999 |
| **Actual body weight (in kg)** | 1.033 | [1.001,1.067] | **.045** |
| **Dosing frequency (Ref. BID)** |  |  |  |
| TID | .000 | [.000, inf] | .999 |
| OD | 1.833 | [.428,7.859] | .414 |
| Every 2 Days | .000 | [.000, inf] | 1.000 |
| **Duration of therapy (days)** | .997 | [.909,1.094] | .952 |

**Table S9: Multivariate Logistic Regression (Out: Failure (Scr increase of 3 times or 75% reduction in CrCL)**

| **Variable** | **ODDS Ratio** | **95% CI** | **P** |
| --- | --- | --- | --- |
| **Gender (Ref. Male)** |  |  |  |
| Female | .058 | [.003,1.020] | **.05** |
| **Concurre antibiotic (2) (Ref. No)** |  |  |  |
| Yes | .312 | [.047,2.070] | .228 |
| **Clcr at the start of vancomycin (ml/min)** | 1.011 | [.994,1.028] | .197 |
| **Actual body weight (in kg)** | 1.047 | [.999,1.096] | **.05** |

**Table S10: Univariate Logistic Regression (Out: Rel replaceme therapy)**

| **Variable** | **ODDS Ratio** | **95% CI** | **p** |
| --- | --- | --- | --- |
| **Age** | 1.026 | [.962,1.094] | .440 |
| **Gender (Ref. Male)** |  |  |  |
| Female | .000 | [.000, inf] | .997 |
| **BMI, (Ref. Underweight)** |  |  |  |
| Normal Weight | .385 | [.022,6.757] | .513 |
| Overweight | .000 | [.000, inf] | .998 |
| Obesity Class I | .000 | [.000, inf] | .998 |
| Obesity Class II | .000 | [.000, inf] | .999 |
| Obesity Class III | .000 | [.000, inf] | .999 |
| **Site of infection, (Ref. Iraabdomil)** |  |  |  |
| Skin | 115391082.013 | [.000, inf] | .999 |
| Pulmory | 1.000 | [.000, inf] | 1.000 |
| Urine | 1.000 | [.000, inf] | 1.000 |
| Other | 75138378.985 | [.000, inf] | .999 |
| **Presence of Bacteria (Ref. No)** |  |  |  |
| Yes | .587 | [.052,6.684] | .668 |
| **Type of pathogen (Ref. CoNS)** |  |  |  |
| E. faecalis | .704 | [.061,8.107] | .778 |
| MSSA | .000 | [.000, inf] | 1.000 |
| E. facium | .000 | [.000, inf] | .999 |
| **Polymicrobic infection? (Ref. No)** |  |  |  |
| Yes | 4.667 | [.407,53.448] | .216 |
| **Concurre antibiotic (1) (Ref. No)** |  |  |  |
| Yes | .062 | [.004,885] | **.004** |
| **Concurre antibiotic (2) (Ref. No)** |  |  |  |
| Yes | 1.125 | [.099,12.842] | .924 |
| **Concurre antibiotic (3) (Ref. No)** |  |  |  |
| Yes | 1.062 | [.093,12.153] | .961 |
| **Hospital length of stay** | 1.013 | [.997,1.029] | .115 |
| **Charlson Comorbidity Index (CCI)** | 1.185 | [.786,1.787] | .417 |
| **10-y survival (%)** | .987 | [.957,1.018] | .402 |
| **Imunno suppress status (Ref. No)** |  |  |  |
| Yes | .000 | [.000, inf] | .998 |
| **Scr level at the start of vancomycin (mcmol/L) ‎** | 1.751 | [.000, inf] | .982 |
| **Clcr at the start of vancomycin (ml/min)** | .365 | [.053,2.525] | .307 |
| **Admission to ICU (Ref. No)** | 74560376.750 | [.000, inf] | .998 |
| Yes |  |  |  |
| **Vancomycin maience dose (Ref. 500 mg)** |  |  |  |
| 1000 mg | 32968864.823 | [.000, inf] | 1.000 |
| 1500 mg | 1.000 | [.000, inf] | 1.000 |
| Others | 89748576.463 | [.000, inf] | .999 |
| **Actual body weight (in kg)** | 1.003 | [.956,1.053] | .896 |
| **Dosing frequency (Ref. BID)** |  |  |  |
| TID | 1.000 | [.000, inf] | 1.000 |
| OD | 156336277.018 | [.000, inf] | .997 |
| Every 2 Days | 1.000 | [.000, inf] | 1.000 |
| **Duration of therapy (days)** | 1.040 | [.963,1.112] | .321 |

**Table S11: Multivariate Logistic Regression (Out: Rel replaceme therapy)**

| **Variable** | **ODDS Ratio** | **95% CI** | **p** |
| --- | --- | --- | --- |
| **Concurre antibiotic (1) (Ref. No)** |  |  |  |
| Yes | .062 | [.004,885] | **.004** |
